# Levels of IgG subclasses 1–4 in BT595, a 10% IVIG: A sub-analysis of a clinical trial

**DOI:** 10.1371/journal.pone.0357472

**Published:** 2026-09-25

**Authors:** Fabian Bohländer, Silke Aigner, Joachim Schütze, Jörg Schüttrumpf, Christiane Staiger

**Affiliations:** Biotest AG, Dreieich, Germany; Bangladesh Council of Scientific and Industrial Research, BANGLADESH

## Abstract

The 10% intravenous immunoglobulin (IVIG) BT595 was evaluated in an open-label, prospective, multicenter clinical trial (Trial 991; NCT02810444) to characterize IgG subclass pharmacokinetics as replacement therapy in patients with primary immunodeficiency (PID). Patients received BT595 at either a 3-week (Q3W) or 4-week (Q4W) dosing interval for approximately 12 months. Secondary endpoints included steady state trough concentrations and pharmacokinetic parameters of IgG subclasses 1–4 and total IgG. IgG subclass trough concentrations were assessed at baseline and at steady state prior to the 7th infusion (Q3W) or 5th infusion (Q4W). Pharmacokinetic parameters at steady state were determined in patients aged ≥6 years using serum samples collected predose and up to 28 days post infusion. At steady state, trough concentrations of total IgG and all IgG subclasses were comparable between BT595 and patients’ previous IVIG therapy, indicating preservation of systemic IgG exposure following treatment switch. IgG subclass trough concentrations were stable across dosing regimens and age categories, with a proportional distribution closely matching physiological IgG composition. IgG1 and IgG2 accounted for the majority of total IgG, whereas IgG3 and IgG4 contributed smaller fractions, consistent with endogenous IgG and published IVIG data. Pharmacokinetic profiles of all IgG subclasses were consistent with those reported for licensed IVIG products. Maximum concentrations occurred rapidly after infusion, and systemic exposure was robust under both dosing regimens. Mean concentrations and C_max_ values were higher with Q3W dosing, while exposure over the dosing interval (AUC_tau_) was lower compared with Q4W dosing due to the shorter interval. Estimated half-life values were within or above ranges reported for other IVIG products, with numerically prolonged persistence of IgG2 and IgG4 under Q4W dosing. Overall, BT595 provides sustained IgG subclass exposure at steady state, supporting its suitability as IVIG replacement therapy in PID under both Q3W and Q4W regimens.

## Introduction

Intravenous immunoglobulin (IVIG) is indicated as a replacement therapy in persons with primary immunodeficiencies (PID). BT595 (Yimmugo®, a registered trademark in the European Union and certain other countries, Biotest AG, Dreieich, Germany) is a highly purified, new 10% IVIG preparation. The manufacturing process comprises four effective virus inactivation / removal steps (i.e., caprylic acid treatment, pH 4 treatment, anion exchange chromatography and 20 nm nanofiltration) resulting in high margins of safety with respect to infectious viruses. In addition, low levels for IgA, IgM and accompanying plasma proteins are also achieved, and the drug product has been shown to have no procoagulant activity [1].

The efficacy, safety, and pharmacokinetics (PK) of BT595 has been assessed in an open-label, prospective, uncontrolled, multicenter Phase III pivotal trial in pediatric and adult patients with PI (EudraCT: 2015-003652-52; NCT02810444). The primary objective was to demonstrate that the rate of acute serious bacterial infections (i.e., the mean number of acute serious bacterial infections per subject-year) was less than 1.0, to provide substantial evidence of efficacy and has been achieved. The secondary objectives of the trial, in addition to further efficacy assessments, were to assess the safety and PK characteristics of BT595. Details on the trial design, eligibility criteria and efficacy and safety results have been published previously [2,3]. Here we report on a sub-analysis of this trial to explore the PK parameters of the IgG subclasses 1‑4 and total IgG for all patients aged 6 to <76 years.

Human IgG is divided into four subclasses, namely IgG1 (60–70%), IgG2 (20–30%), IgG3 (5–8%) and IgG4 (2–6% of total IgG). The IgG subclasses share a common overall structure (>90% identical amino acids) but differ in their constant (Fc) regions, hinge regions, and binding affinities to Fcγ receptors and complement factors [4]. Due to these differences also the effector functions of IgG subclasses differ:

IgG1 and IgG3 are the most efficient in mediating classical effector functions: They bind well to Fcγ receptors on phagocytes and NK cells, promoting antibody-dependent cellular phagocytosis (ADCP) and antibody-dependent cellular cytotoxicity (ADCC) [5]. They are also potent activators of the complement system via C1q binding, contributing to complement-mediated lysis or opsonization. Among them, IgG3 often has the highest complement activity, largely because of its long hinge region allowing better C1q engagement [4].

IgG2 has a more restricted effector profile. It interacts less strongly with most Fcγ receptors and is relatively weak in complement activation, thus being less effective in ADCC or complement-dependent cytotoxicity (CDC) under many circumstances. IgG2 responses are often elicited in response to carbohydrate or polysaccharide antigens (e.g., bacterial capsular polysaccharides) [6].

IgG4 is unique in having low or negligible ability to activate complement, and reduced capacity to bind activating Fcγ receptors [7]. IgG4 can undergo “Fab-arm exchange,” meaning half-antibodies can swap arms, leading to functional monovalency and bispecific antibodies. Because of its subdued effector functions, IgG4 is often considered anti-inflammatory or “blocking” in allergen or immune tolerance settings (e.g., allergen immunotherapy) [8].

The PK properties of the IgG subclasses are driven by their ability to bind to the neonatal Fc receptor (FcRn), which contributes to their half-life, recycling in serum, and transport across the placenta [4]. Whereas the half-life of IgG1, IgG2 and IgG4 is about 21–22 days in human serum due to binding to FcRn, IgG3 only shows a half-life of 7 days, because of impaired binding to this receptor [9,10].

In summary, the 4 IgG subclasses allow the immune system to tailor humoral responses depending on antigen type, need for effector activation, and balancing immunity vs. tolerance. Alterations in human IgG subclass distribution are linked to characteristic disease patterns: elevated IgG1 and IgG3 often reflect chronic antigen stimulation, selective IgG2 deficiency is linked to recurrent infections by encapsulated bacteria, and increased IgG4 characterizes tolerance-driven responses and the entity IgG4-related disease [4,7,11].

A distribution of the 4 IgG subclasses, similar to the natural distribution, is therefore important for the quality, efficacy and safety of our medicinal product BT595.

## Materials and methods

### Trial design and patient population

Trial 991 was an open-label, prospective, uncontrolled, multicenter Phase III pivotal trial (EudraCT: 2015-003652-52/17046; NCT02810444). Trial protocol and statistical analysis plan can be accessed at clinicaltrials.gov under the related NCT. The trial was conducted in accordance with the International Conference on Harmonization Good Clinical Practice (ICH-GCP) guidelines and the Declaration of Helsinki. The trial protocol and all other relevant documents were reviewed and approved by the competent local Independent Ethics Committees. Written informed consent was obtained from all patients or patients’ parents / legal guardians. Trial characteristics, overview of patient population and important efficacy and safety results were published for the overall cohort and for the pediatric subgroup [2,3].

**Fig 1:**
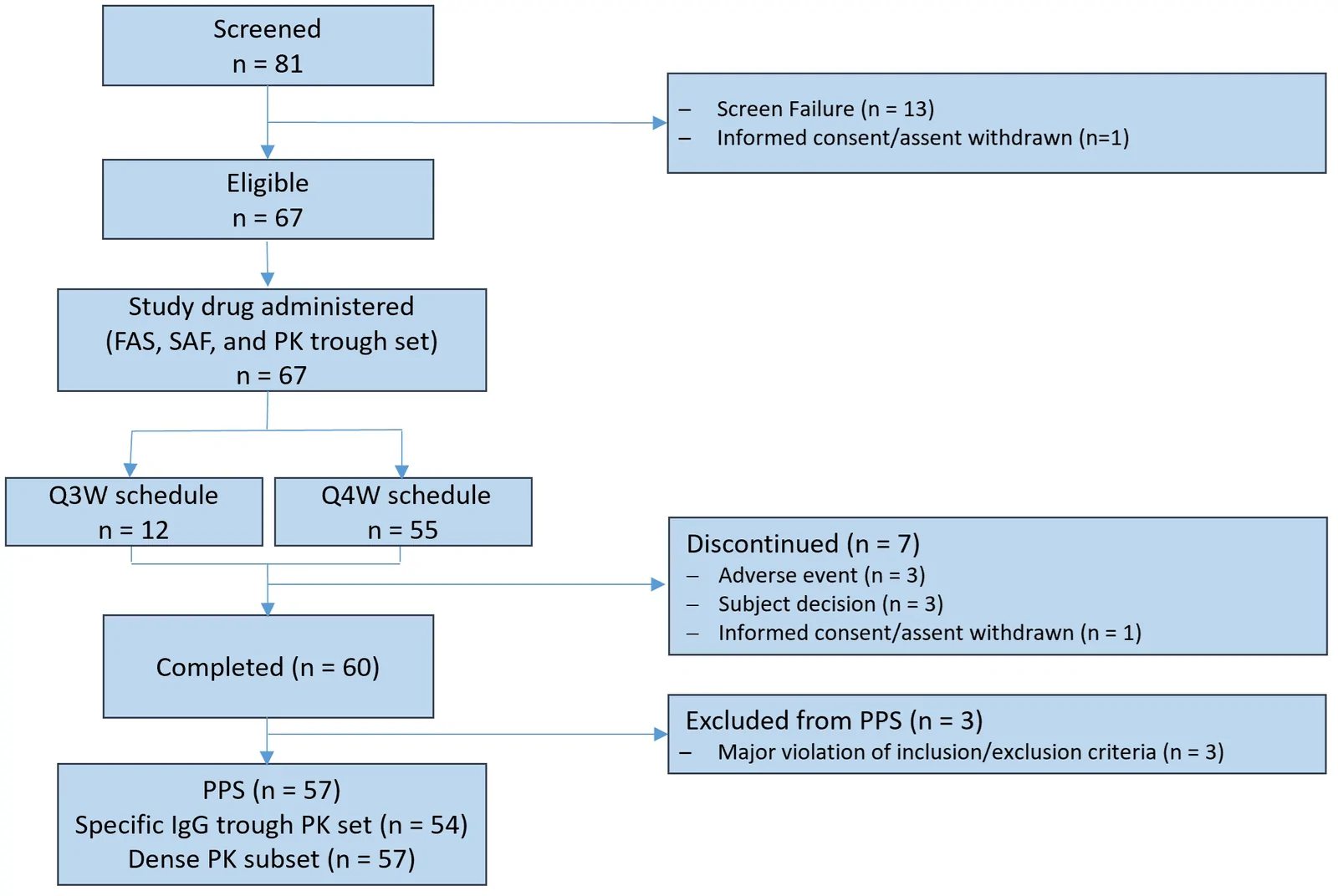
Patient disposition. Abbreviations: FAS, full analysis set; n, number of patients in a specified category; PK, pharmacokinetics; PPS, per protocol set; Q3W, every 3 weeks; Q4W, every 4 weeks; SAF, safety analysis set.

In the trial, all 67 patients (49 adults, 18 children) received BT595 in doses between 0.2 and 0.8 g/kg body weight for approximately 12 months at intervals of 3 (Q3W) or 4 (Q4W) weeks (Fig 1).

IgG subclasses 1‑4 trough levels were measured at baseline and before the 7th/5th infusion of the Q3W/Q4W schedule, respectively. For PK parameters at steady state, serum samples from patients ≥6 years were analyzed at the time points: predose, 10–30 minutes, 4, 24 hours, and 4, 7, 14, 21, 28 days post infusion.

The PK trough set included all 67 subjects, for whom ≥1 trough concentration of total IgG were available. Data on IgG subclasses 1‑4 were available for 64 of these patients. Dense PK subset includes all subjects who received all planned doses and for whom ≥1 concentration of total IgG and IgG1-4 was available, this included 57 patients aged 6 to <76 years (Table 1).

**Table 1:** Demographics at baseline for PK trough and dense PK subsets in trial 991.

Characteristics	Q3W	Q4W	Overall
**PK trough Set**			
*n*	12	55	67
*Age (years)*			
Mean (SD)	36.1 (25.17)	34.3 (18.91)	34.6 (19.97)
Median	37.0	37.0	37.0
Min – Max	3 - 69	2 - 74	2 - 74
*Gender*			
Male	5 (41.7)	32 (58.2)	37 (55.2)
Female	7 (58.3)	23 (41.8)	30 (44.8)
**Dense PK Set**			
*n*	10	47	57
*Age (years)*			
Mean (SD)	40.8 (24.62)	37.9 (17.51)	38.4 (18.72)
Median	50.5	39.0	40.0
Min – Max	6 - 69	10 - 74	6 - 74
*Gender*			
Male	4 (40.0)	26 (55.3)	30 (52.6)
Female	6 (60.0)	21 (44.7)	27 (47.4)

Abbreviations: n, number of patients in a specified category; max, maximum; min, minimum; PK, pharmacokinetic(s); Q3W, 3-week schedule; Q4W, 4-week schedule; SD, standard deviation.

### Determination of IgG1-4 subclasses serum levels

Analysis of total IgG, IgG1, IgG2, IgG3, IgG4 in human serum samples was done by in house developed enzyme-linked immunosorbent assays (ELISA). All analytical methods have been successfully validated according to current bioanalytical guidelines in the Good Laboratory Practice (GLP) testing facility. Besides absolute serum concentrations, each individual concentration (g/L) were transformed to relative proportion of the total of the 4 subclasses (percent of the total).

### Pharmacokinetic analysis

Analyses of IgG1-4 trough levels (before each infusion), concentration versus time profiles, and non-compartmental PK analyses (NCAs) were performed to assess the PK profile for total IgG and IgG subclasses 1–4.

PK parameters were derived from NCA using Phoenix WinNonLin 6.3. Actual sampling times for each subject were used. The following PK parameters were determined: maximum concentration (C_max_), time to reach maximum concentration (t_max_), trough concentration (C_trough_), area under the concentration-time curve (AUC) calculated from start to the end of dosing interval (AUC_tau_), terminal elimination half-life (t_1/2_) and AUC calculated from time zero extrapolated to infinity (AUC_(0-inf)_).

### Statistical analysis

For this IgG subclass subanalysis, statistical analyses were primarily descriptive. Trough concentrations, relative subclass percentages, concentration-time data, and NCA-derived PK parameters were summarized by dosing schedule using the number of subjects (n), mean (SD), median, minimum, and maximum. Analyses used observed concentrations and actual sampling times; missing concentrations were not imputed. Terminal-phase PK parameters were summarized only when the λz regression fulfilled the predefined reliability criterion (adjusted R2 ≥ 0.8500). The number of subjects contributing to each PK parameter is shown as n in the respective tables. The exploratory equivalence comparison of steady-state trough concentrations is described below.

### Exploratory equivalence testing

Exploratory equivalence testing by ANOVA, based on guidance for bioequivalence testing by EMA and FDA [12,13], was performed as a supportive intra-subject comparison of steady state trough levels of the IgG subclasses 1‑4 between BT595 and previous IVIG reference therapy. Log-transformed values for the parameter Css/D (dose-normalized steady state trough concentration) for the IgG subclasses 1–4 were compared with treatment as fixed effect and subject as random effect. The following hypotheses were tested for Css (steady state trough concentration) and Css/D values, to compare BT595 (T = test) and previously administered IVIG (R = reference). μT and μR denote the corresponding model-based geometric means of the analyzed trough parameter for test (μT) and reference (μR):

*H01: μT/μR ≤ 80% vs. HA1: μT/μR > 80% and H02: μT/μR ≥ 125% vs. HA2: μT/μR < 125%.*

If the 90% CI for the geometric mean ratio (GMR) fell within 80% to 125% for any given comparison, the null hypotheses, that is H01 and H02, had to be rejected, with the conclusion that BT595 was equivalent to the previously administered IVIG for the respective parameter.

## Results

Patients were enrolled from 4 October 2016, and the last patient completed on 1 April 2020. Patients were treated in 17 sites on three continents [2]. 49 patients (73.1%) were adults, including 5 geriatric patients (≥65 years), and 18 subjects (26.9%) were pediatric patients (2–16 years).

Baseline demographics for the PK trough set and the dense PK set are presented in Table 1. Age of patients ranged from 2–74 years with a mean of 34.6 years in the PK trough set (n = 67), as well as from 6–74 years, mean 38.4 years, in the dense PK set (n = 57). Patients sex was equally distributed between male and female. There were no notable differences between 3-week and 4- week schedule.

### Comparison of IgG1-4 trough levels with previous IVIG therapy (baseline)

**Fig 2:**
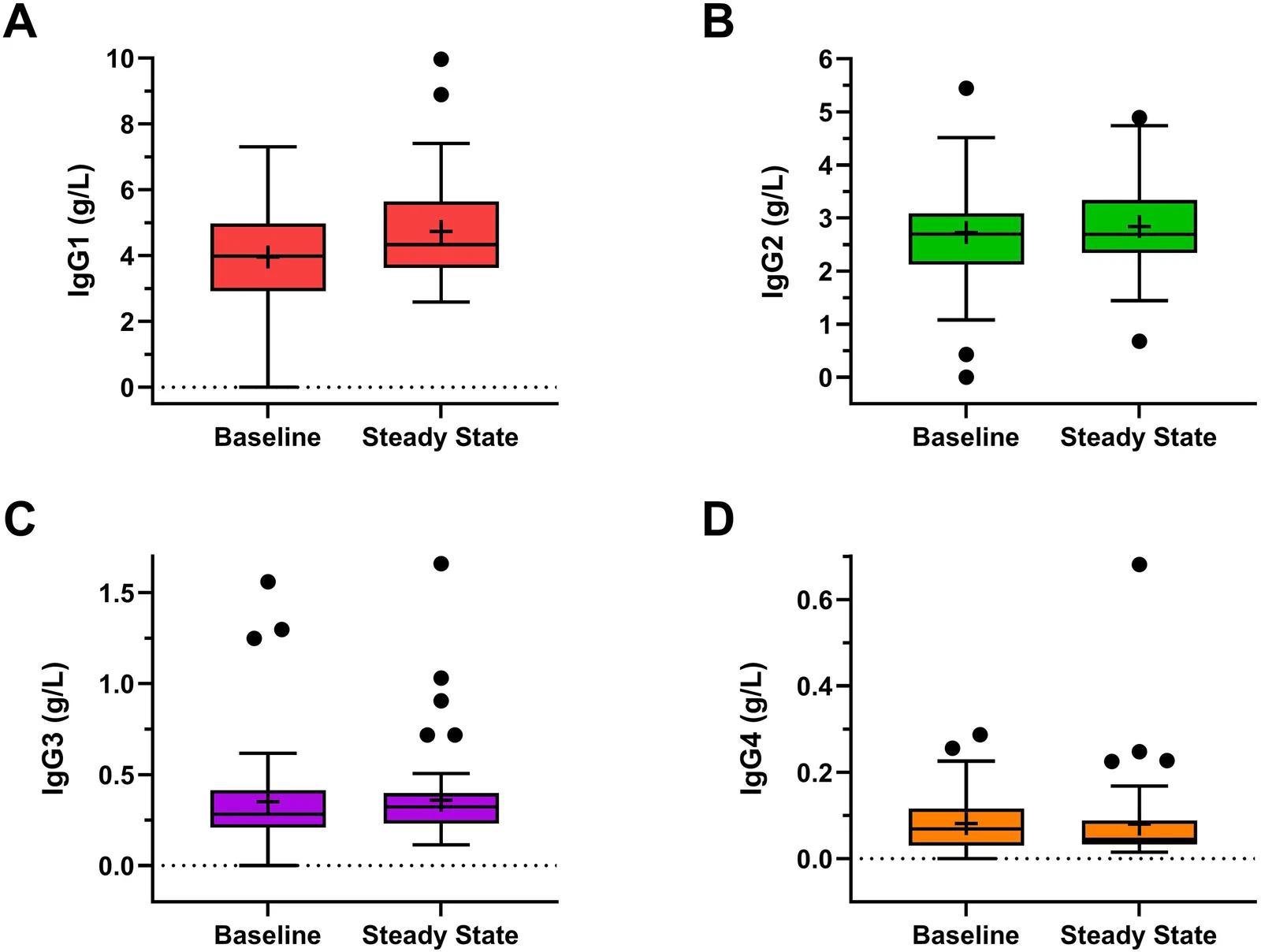
Trough levels of IgG1 (A), IgG2 (B), IgG3 (C) and IgG4 (D) at Steady State for BT595 and Previous Reference Immunoglobulin (Baseline) – PK trough set (N = 64, Age 6 to <76 Years). The cross symbol represents the arithmetic mean and the horizontal line represents the median value. The box represents the 1st and 3rd quartile (25th and 75th percentile). The whiskers represent the Min – Max range, or the 1.5 fold IQR in case of outliers (beyond the 1.5 fold IQR, shown as circles). Abbreviations: IgG, immunoglobulin G, IQR, interquartile range (25% to 75% percentile); Min, minimum; Max, maximum.

The trough levels of IgG subclasses 1‑4 at steady state are shown by box plot presentations of the data from 64 subjects aged 6 to <76 years (PK trough set) in Fig 2. IgG1-4 trough levels did not differ notably between BT595 and subjects’ previous IVIG reference therapy.

In addition, no clinically relevant differences of the steady state IgG subclass trough levels between BT595 and subjects’ previous IVIG reference therapy were noted in the different age groups (6 to <12 years, 12 to <17/18 years, 17/18 to <76 years, 17/18 years to <65 years, and ≥65 years, data not shown). Also, no relevant differences were observed between and within Q3W and Q4W schedule (data not shown).

Exploratory equivalence testing by ANOVA, based on guidance for bioequivalence testing, was performed to compare steady state trough levels of the IgG subclasses 1‑4 between BT595 (test) and previous IVIG reference therapy. Results were interpreted descriptively as this trial was not designed as a formal equivalence or bioequivalence study.

For the Q4W schedule group overall, the analyses indicated no clinically relevant differences between the steady state IgG subclass trough levels for BT595 and those for the previous IVIG reference therapy. Trough levels were equivalent for IgG2 and IgG3 (90% CI for the geometric mean ratio (GMR) [x 100] within the range of 80% to 125%) but were higher for IgG1 (90% CI for the GMRx100: 107.7 to 138.2%) and were lower for IgG4 (90% CI for the GMRx100: 65.8 to 91.7%) (Table 2).

**Table 2:** Exploratory comparisons (ANOVA) of trough levels of IgG subclasses 1‑4 at steady state to assess the equivalence of BT595 and previous reference immunoglobulin – PK trough set.

N with data at baseline / steady state	Q3W (N = 5 / 10 patients)			Q4W (N = 41 / 45 patients)		
	LS Mean (SE)		GMR x 100 [90% CI]	LS Mean (SE)		GMR x 100 [90% CI]
	Previous IVIG (Baseline)	BT595 (Steady State)		Previous IVIG (Baseline)	BT595 (Steady State)	
IgG1	1.661 (0.102)	1.644 (0.098)	98.3 [89.9, 107.5] p = 0.709	1.278 (0.063)	1.477 (0.061	122.0 [107.7, 138.2] **p = 0.011**
IgG2	1.355 (0.113)	1.251 (0.097)	90.2 [75.5, 107.7] p = 0.281	0.931 (0.054)	0.936 (0.052)	100.5 [92.3, 109.3] p = 0.925
IgG3	−1.158 (0.226)	−1.176 (0.202)	98.3 [71.8, 134.4] p = 0.910	−1.322 (0.106)	−1.225 (0.105)	110.2 [97.5, 124.6] p = 0.188
IgG4 ^a^	−2.206 (0.246)	−2.505 (0.200)	74.2 [47.2, 116.6] p = 0.232	−2.710 (0.121)	−2.962 (0.117)	77.7 [65.8, 91.7] **p = 0.015**

Abbreviations: ANOVA, analysis of variance; CI, confidence interval; GMR, geometric mean ratio; IgG, immunoglobulin G, IVIG, immunoglobulin for intravenous administration; LS mean, least‑squares mean of log-transformed PK parameter; N, number of patients; Q3W, 3-week schedule; Q4W, 4-week schedule; SE, standard error.

^a^N with data at baseline / steady state**:** Q4W: 40 / 45 subjects.

For the GMR x 100, estimates of the mean and the CI limits for the ln values were exponentiated and multiplied by 100. The ANOVA was performed on log-transformed dose-normalized steady-state trough concentrations (Css/D), with treatment as a fixed effect and subject as a random effect. Equivalence criterion: 90% CI for GMR within 80% to 125%. P values are nominal and descriptive; no multiplicity adjustment was applied.

These analyses have to be interpreted with caution due to the limited number of subjects with data (n = 41) in the Q4W schedule. Thus, exploratory equivalence was shown for IgG2 and IgG3 in the Q4W group. The Q3W schedule group and the pediatric subgroups were too small (≤10 subjects) for any meaningful conclusions.

### Relative distribution of IgG1-4 and comparison to normal human values

**Fig 3:**
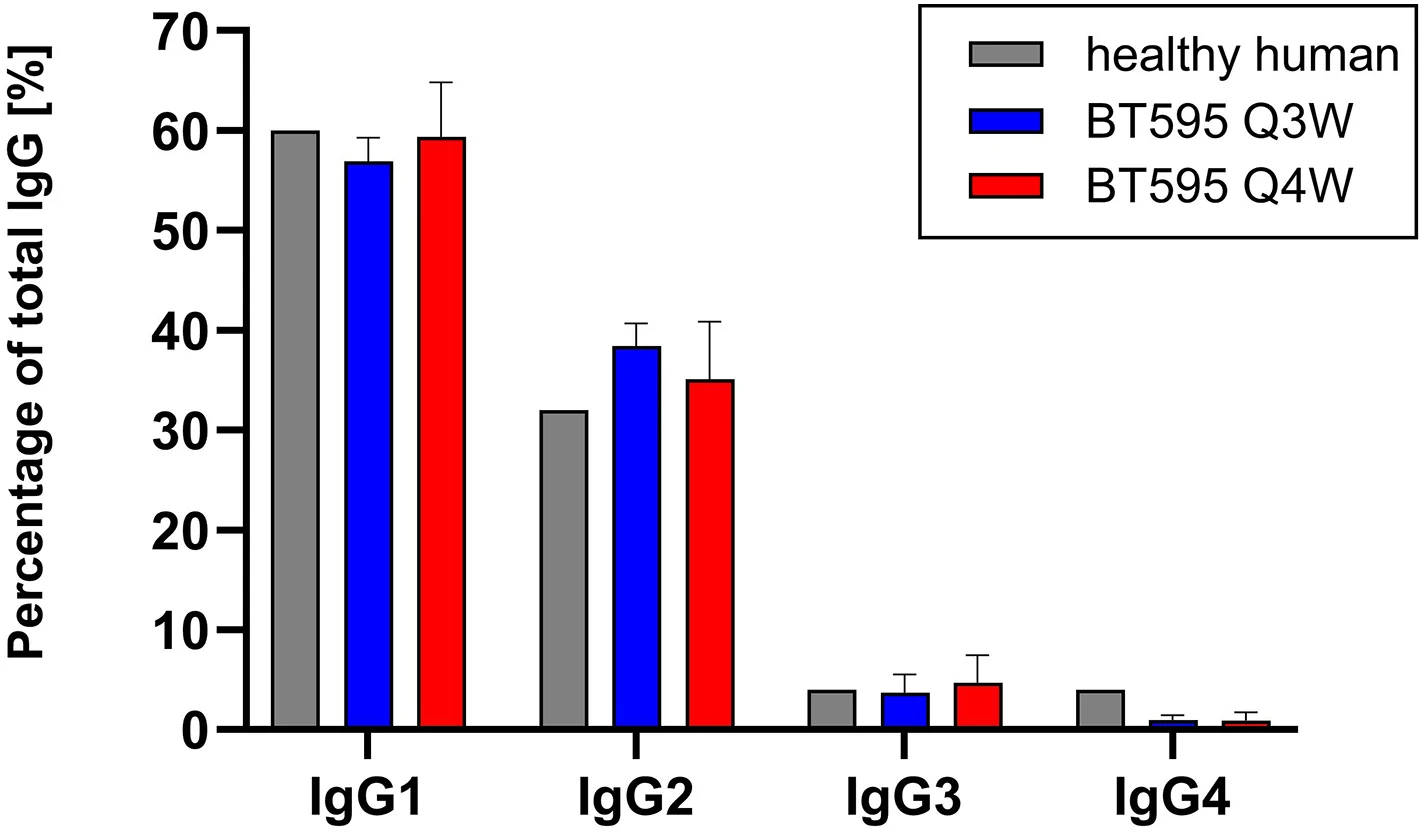
Relative percentage of IgG subclasses 1-4 in total IgG trough levels at BT595 steady state – PK trough set (N = 64, Age 6 to <76 Years). Abbreviations: IgG1-4, immunoglobulin G subclasses 1-4; Q3W, 3-week schedule; Q4W, 4-week schedule; PK, pharmacokinetic.

There were no clinically relevant differences in the relative percentage of IgG subclasses 1‑4 in trough levels between baseline and BT595 steady state for all subjects ≥6 years in either treatment schedule. Overall, IgG subclasses 1 and 2 were the most abundant (accounting for >50% and >30% of the total IgG sample, respectively), whereas IgG subclasses 3 and 4 each accounted for <5% of the total IgG (Fig 3 and Table 3). There were also no clinically relevant differences between age categories (6 to <12 years, 12 to <17/18 years, 17/18 to <76 years, 17/18 years to <65 years, and ≥65 years) (data not shown).

**Table 3:** Relative percentage and Ctrough levels of IgG subclasses 1‑4 at BT595 steady state – PK trough set (N = 64, Age 6 to <76 years).

		Q3W (n = 11) mean (SD)	Q4W (n = 53) mean (SD)	Reported adult ranges^1^ median (ranges)
IgG1	C_trough_ (g/L)	5.41 (1.594)	4.58 (1.537)	6.85 (4.45–9.76)
	% of total	56.89 (2.432)	59.35 (5.494)	60 (43-75)
IgG2	C_trough_ (g/L)	3.66 (1.088)	2.66 (0.663)	4.81 (2.07–8.57)
	% of total	38.42 (2.286)	35.09 (5.755)	32 (16-48)
IgG3	C_trough_ (g/L)	0.36 (0.254)	0.36 (0.253)	0.29 (0.08–0.8)
	% of total	3.70 (1.820)	4.67 (2.751)	4 (1.7-7.5)
IgG4	C_trough_ (g/L)	0.1 (0.064)	0.08 (0.105)	0.46 (0.05–1.54)
	% of total	0.99 (0.466)	0.9 (0.818)	4 (0.8-11.7)
Total IgG	C_trough_	10.03 (2.276)	8.27 (2.341)	12.41 (6.65-20.67)

^1^C_trough_ levels [14], % of total [4,5] Abbreviations: IgG, immunoglobulin G; N, number of subjects; Q3W, 3-week schedule; Q4W, 4-week schedule; SD, standard deviation.

The distribution of IgG1-4 is similar to that in healthy humans, with only slightly higher IgG2 (38.4% vs. 32%) in Q3W schedule and slightly lower IgG4 (1% vs. 4%) for Q3W-and Q4W schedule than reported median levels in literature (Fig 3 and Table 3) [4]. Of note the measured IgG1-4 levels were within the published ranges [5]. When comparing the absolute IgG1-4 trough levels with literature, slightly lower values as reported in healthy human were achieved with BT595 therapy at steady state (Table 3), although these values are within the published ranges [14].

### Concentration vs. time profile of IgG1-4 and key PK parameters

**Fig 4:**
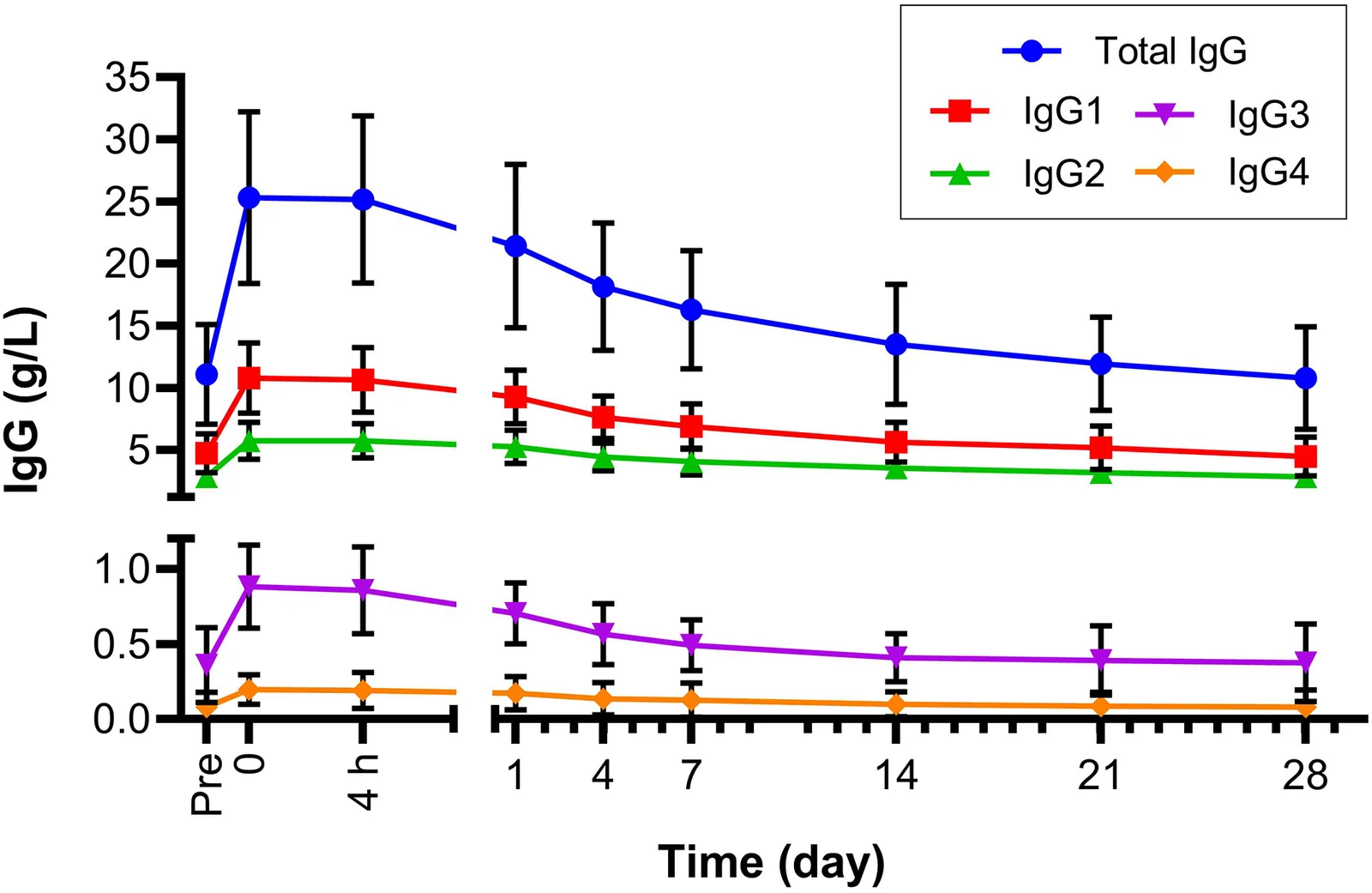
Concentration versus time profiles for IgG subclasses 1-4 at steady state – dense PK subset (N = 57). Abbreviations: IgG1-4, immunoglobulin G subclasses 1-4; PK, pharmacokinetic.

Fig 4 presents the mean (SD) concentration versus time profiles at steady state for IgG subclasses 1‑4 and total IgG. Pharmacokinetic (PK) parameters for total IgG and IgG subclasses at steady state following BT595 administration were evaluated in a dense PK subset (N = 57) receiving either a Q3W (n = 10) or Q4W (n = 47) dosing regimen (Table 4).

**Table 4:** Key PK parameters for total IgG and IgG subclasses 1‑4 at steady state after infusion of BT595 – dense PK subset (N = 57).

	Q3W, N = 10		Q4W, N = 47	
	n	Mean (SD) or Median (Min – Max)	n	Mean (SD) or Median (Min – Max)
*C*_*max*_ *(g/L)*				
Overall	10	30.0 (7.65)	45	26.5 (6.08)
IgG1	10	12.9 (3.47)	45	10.8 (2.49)
IgG2	10	7.3 (1.78)	45	5.7 (1.25)
IgG3	10	1.1 (0.43)	45	0.9 (0.23)
IgG4	10	0.3 (0.11)	45	0.2 (0.12)
*t*_*max*_ *(day)*				
Overall	10	0.22 (0.06–0.28)	45	0.20 (0.06–7.03)
IgG1	10	0.15 (0.08–0.27)	45	0.18 (0.05–1.11)
IgG2	10	0.25 (0.09–1.08)	45	0.11 (0.06–4.01)
IgG3	10	0.19 (0.08–1.09)	45	0.11 (0.00–21.3)
IgG4	10	0.13 (0.08–0.27)	45	0.10 (0.05–6.86)
*AUC*_*tau*_ (day*g/L)				
Overall	10	374.3 (110.69)	42	398.5 (99.27)
IgG1	10	161.1 (43.22)	42	167.6 (39.49)
IgG2	10	103.0 (25.77)	42	100.0 (20.00)
IgG3	10	11.0 (4.36)	41	12.2 (4.09)
IgG4	10	3.0 (1.48)	41	2.6 (1.34)
*AUC*_*0-inf*_ (day*g/L) ^*a*^				
Overall	7	761.2 (158.07)	25	817.3 (259.82)
IgG1	8	372.4 (157.57)	31	354.6 (115.30)
IgG2	8	319.3 (139.22)	36	298.1 (225.27)
IgG3	9	27.42 (23.09)	19	23.32 (10.86)
IgG4	8	7.736 (6.07)	36	8.436 (14.66)
*t*_*1/2*_ *(day)* ^*a*^				
Overall	7	24.15 (5.899)	25	31.13 (12.899)
IgG1	8	26.64 (7.292)	31	30.92 (11.481)
IgG2	8	35.28 (11.841)	36	46.84 (35.545)
IgG3	9	26.34 (13.174)	19	29.20 (11.294)
IgG4	8	26.01 (15.365)	36	44.42 (71.513)

Abbreviations: AUC_tau,_ area under the concentration-time curve calculated from start to end of the dosing interval; AUC_0-inf,_ area under the concentration-time curve extrapolated to infinity; C_max_, maximum concentration; IgG, immunoglobulin G; Max, maximum; Min, minimum; N, total subjects; n, number of subjects with data; Q3W, 3‑week schedule; Q4W, 4-week schedule; SD, standard deviation, t_max_, time to reach the maximum concentration; t_1/2_, terminal elimination half-life.

^a^Values with R2 adjusted <0.8500 were excluded. For AUC_0-inf_ and t_1/2_, n is lower because terminal-phase parameters were calculated only when sufficient concentration-time data were available. Statistical/methodological note: PK parameters are descriptive. Actual sampling times were used; C_max_ and t_max_ were read from observed data, and AUC values were calculated using the linear/log trapezoidal rule. No PK imputation was applied.

Across both schedules, serum concentrations of total IgG and IgG subclasses increased rapidly after infusion, showing the typical profile known for intravenous application of IVIG with a sharp increase after end of infusion and with a slow decline until day 28. Consequently, tmax occurs within the first 0.10 to 0.25 days (2.4 to 6 hours) (Table 4).

Similar IgG1-4 concentration time profiles were observed for each treatment schedule. Consistent with the total IgG data, mean and maximum concentrations were lower in the Q4W schedule group than in the Q3W schedule group for all 4 subclasses and at all time points assessed.

Mean C_max_ values for total IgG were slightly higher in the Q3W cohort (30.0 g/L) compared with the Q4W cohort (26.5 g/L), consistent with the shorter dosing interval and higher baseline levels prior to re-dosing. This pattern was similarly observed for all IgG subclasses, where Q3W dosing yielded moderately higher maximum concentrations. Coefficients of variation for C_max_ were generally between 20–30% for total IgG, IgG1, and IgG2, with higher variability observed for IgG3 and IgG4.

Although steady state AUC over the dosing interval (AUC_tau_) was slightly lower in the Q3W schedule group due to the 7‑day shorter dosing interval (mean 374.3 day·g/L for Q3W vs 398.5 day·g/L for Q4W), a similar overall exposure was observed. Subclass AUC patterns reflected relative abundance, with IgG1 accounting for the majority of subclass exposure, followed by IgG2, IgG3 and IgG4.

For subjects with reliable terminal phase characterization, AUC_0-inf_ values suggested slightly higher cumulative exposure with the Q4W regimen for total IgG (mean 817.3 vs 761.2 day·g/L), though high variability. Subclass AUC_0-inf_ values followed consistent patterns, with IgG1 and IgG2 contributing the largest share of exposure across both dosing schedules.

Estimates of terminal half-life (t_1/2_) reflected typical IgG kinetics. In the Q3W group, mean t_1/2_ for total IgG was 24.2 days, consistent with known IgG turnover rates. The Q4W group demonstrated a somewhat longer mean half-life (31.1 days), although variability was high. Among subclasses, IgG1 and IgG3 showed half-lives in the 26–31-day range in both regimens. IgG2 and IgG4 exhibited greater between-subject variability, with IgG2 showing the longest mean half-life in both schedules (35.3 days Q3W; 46.8 days Q4W) (Table 4).

## Discussion

Intravenous immunoglobulins (IVIGs), consisting of pooled polyclonal human IgG, have been used for several decades as replacement therapy in patients with primary immunodeficiency (PID) and other immune-mediated diseases [15,16]. The pharmacokinetic (PK) properties of total IgG administered via IVIG are well characterized and extensively described in the literature, reflecting regulatory requirements for product authorization. Both EMA and FDA guidelines mandate PK characterization of total IgG as part of clinical development programs for IVIG products in PID populations [17,18].

Importantly, while these regulatory guidelines recommend measurement of IgG subclasses at steady state, they do not define formal target concentrations for individual IgG subclasses. This is notable given that IgG subclasses differ in their biochemical properties, Fc-mediated functions, and immunological roles, which may have implications for clinical efficacy in antibody replacement therapy [4]. Despite this relevance, detailed subclass-specific PK data for IVIG products remain limited. Available information largely originates from historical studies, and there is a scarcity of subclass PK data for more recently developed IVIG preparations.

Against this background, the present trial provides a comprehensive characterization of IgG subclass PK properties (IgG1–4) for BT595 under Q3W and Q4W dosing regimens. By systematically evaluating subclass concentration–time profiles, trough levels, maximum exposure, systemic exposure, and half-life, this work adds contemporary data to an underrepresented area of IVIG pharmacology and enables contextualization against both established IVIG products and endogenous IgG physiology.

### Comparison to other IVIGs in literature

The PK profile of BT595 was evaluated for IgG subclasses IgG1–4 at steady state under 3-week and 4-week dosing regimens and compared with published IVIG data [19–26]. While direct cross-study comparisons are inherently limited by differences in trial design, dosing intervals, analytical methods, and reported PK parameters, several observations can be made.

### Serum concentration–time profiles

The serum concentration–time profile of BT595 is consistent with that reported for licensed IVIG products administered intravenously at steady state in PID populations [25,27–31]. Administration of BT595 resulted in a rapid increase in total IgG serum concentrations followed by a characteristic bi-phasic decline. This profile reflects immediate systemic availability after IV infusion, an initial distribution phase into lymphatic and extracellular compartments (α-phase), and a subsequent slower elimination phase (β-phase), as described in the literature [27]. The same kinetic pattern was observed across all IgG subclasses in this trial, with differences in absolute concentrations reflecting their natural distribution within total IgG.

### Trough concentrations (C_trough_)

Maintenance of adequate IgG trough concentrations is a central determinant of IVIG therapy performance. Regulatory and clinical guidance emphasize achieving sufficient total IgG trough levels to reduce infection risk in PID patients. The EMA core SmPC guideline for human normal immunoglobulin recommends achieving a total IgG trough concentration of at least 6 g/L prior to the next infusion, with higher trough values considered beneficial depending on clinical context [17]. FDA guidance does not define a numeric threshold but requires trial protocols to specify and justify minimum acceptable trough levels in relation to clinical outcomes [18]. Neither guideline establishes target concentrations for individual IgG subclasses, although measurement at steady state is recommended.

Literature reviews and meta-analyses indicate that total IgG trough concentrations of ≥5 g/L have traditionally been considered sufficient for clinical benefit, while more recent clinical experience supports higher trough levels in the range of approximately 7–10 g/L to reduce infection rates, including pneumonia, particularly in patients with more severe disease [32–34]..

In this context, BT595 achieved mean total IgG trough concentrations of approximately 10.0 g/L under Q3W dosing and 8.3 g/L under Q4W dosing, exceeding minimum guideline thresholds and aligning with ranges associated with improved clinical outcomes. These values are consistent with pre-infusion or Cmin concentrations reported for established IVIG products [22,23] and numerically higher than trough levels reported in earlier datasets such as Mankarious et al. [19], reflecting differences in dosing strategies and contemporary clinical practice.

At the subclass level, BT595 demonstrated stable trough concentrations with a proportional distribution closely mirroring physiological IgG subclass composition, consistent with published IVIG data [19,22,23,26]. Absolute IgG1 and IgG2 trough concentrations were comparable to, and in some cases numerically higher than, those reported in contemporary studies, with IgG1 troughs typically around 5–6 g/L and IgG2 around 3–4 g/L [23,26]. Under Q3W dosing, BT595 values were toward the upper end of these ranges.

IgG3 and IgG4 trough concentrations were low in absolute terms, as expected given their smaller fractional contribution. Nevertheless, BT595 IgG3 and IgG4 trough levels were comparable to those reported in the literature and showed no evidence of disproportionate depletion. IgG4 concentrations remained within the typical range representing less than 1% of total IgG at trough [22,23,26].

The comparison of trough IgG concentrations during BT595 treatment with baseline values obtained under patients’ prior IVIG therapy demonstrated no clinically meaningful differences in exposure to total IgG or IgG subclasses. Median concentrations and distribution ranges were preserved following the switch to BT595, supporting pharmacokinetic properties similar to established IVIG products [20,22,23,25,26]. Taken together, these results support that treatment with BT595 maintains IgG and IgG subclass trough concentrations comparable to established IVIG replacement therapy, without evidence of reduced exposure following treatment switch, and are consistent with the maintenance of guideline-relevant total IgG trough levels discussed above.

### Maximum concentration (C_max_), Time to maximum concentration (t_max_), and area under the concentration-time curve (AUC)

Maximum serum IgG concentrations (C_max_) occurred rapidly after infusion, with median t_max_ values within the expected range (2–7 hours) for intravenously administered immunoglobulins and comparable to published IVIG studies [22,23,25]. For IgG1 and IgG2, BT595 C_max_ values were comparable to or numerically higher than those reported for both earlier and more recent IVIG formulations, while IgG3 and IgG4 maximum concentrations were similar across products [21–23,25].

Systemic exposure assessed by AUC over the dosing interval was robust with BT595 and fell within the upper range of published values, particularly for IgG1 and IgG2. Although reported AUC values vary substantially across studies due to methodological differences (e.g., steady state versus interval-based analysis and differing integration windows) [20–22,24,25], BT595 consistently demonstrated proportional subclass exposure consistent with physiological IgG composition and no evidence of reduced exposure for any subclass.

### Half-life

Estimated half-life values for IgG subclasses tended to be shorter under Q3W compared with Q4W dosing, likely reflecting the shorter dosing interval and incomplete characterization of the terminal phase before the next infusion. Under Q4W dosing, BT595 half-life values for total IgG and IgG1 were within the range reported for established IVIG products (~30–38 days).Further IgG2 and IgG4 demonstrated numerically longer half-life estimates under Q4W dosing compared with several published studies [20–23,25], although inter-individual variability was high. IgG3 half-life followed the expected pattern of being shorter than other subclasses (see also discussion below) and was comparable to literature values.

Overall, in descriptive cross-study comparisons, the PK profile of BT595 is consistent with that of other licensed IVIG products, with comparable or higher maximum exposure and systemic exposure, and evidence of sustained persistence of clinically relevant subclasses under monthly dosing. While these observations should be interpreted cautiously due to variability and cross-study methodological differences, they may indicate a pharmacokinetic characteristic of BT595, particularly in the context of maintaining protective IgG levels over extended dosing intervals.

### Comparison to endogenous IgG

The primary objective of IVIG replacement therapy in PID and secondary immunodeficiency (SID) is to restore natural IgG-mediated immune functions, which requires both adequate concentrations and a physiological distribution of IgG subclasses. The IgG1–4 trough concentrations observed with BT595, although slightly lower than median values reported for healthy adults, fall within established reference ranges [14,35,36]. Reported reference intervals are broad due to population variability, ethnicity, age, and assay-related differences [14]. Age did not explain the slightly lower trough concentrations observed in this trial, as only minimal differences were seen across age groups (data not shown).

The relative abundance of IgG subclasses with BT595 was consistent with physiological distributions reported in healthy adults [4,5], indicating that replacement therapy with BT595 provides a subclass composition compatible with a functional antibody-mediated immune response. Minor discrepancies between the summed subclass concentrations and total IgG values likely reflect methodological differences between assays used for total IgG and subclass quantification, e.g., nephelometry vs. turbidimetry).

Estimated half-life values for BT595 IgG subclasses (as well as in reference IVIG in literature) were higher than those reported for endogenous IgG, particularly for IgG3. It showed a longer persistence than the ~ 7-day half-life reported for endogenous IgG3. Differences between endogenous and IVIG-derived IgG half-life estimates are well documented and attributed to methodological factors, patient-related variability in the intrinsic production and catabolism of IgG/IgG-subclasses, possible physicochemical alterations during IVIG manufacturing or radiotracer labeling, and assumptions inherent in non-compartmental analysis [27,34,37,38]. Allotypic variation in IgG3 affecting FcRn binding has also been proposed as a potential contributor to prolonged half-life in IVIG preparations [4], although no direct data are available for BT595.

As for other drugs, the half-life of IgG is derived from the terminal phase of the concentration–time profile. However, for IgG this terminal phase is often dominated by stable baseline concentrations that show minimal change over time rather than a true elimination phase. Consequently, continued sampling below baseline without applying baseline correction can result in an overestimation of IgG half-life and unrealistically long half-life values [34].

### Clinical relevance

Alterations in the distribution of human IgG subclasses are associated with characteristic disease patterns. Accordingly, the provision of physiologically balanced levels of IgG subclasses may contribute to normalization of immune function by supplying patients with the antibodies required to prevent or control infections. Such balance enables the immune system to appropriately tailor humoral responses according to antigen type, the requirement for effector function activation, and the need to maintain an equilibrium between protective immunity and immune tolerance. Consequently, an IgG subclass distribution that closely resembles the natural physiological profile is considered important for the quality, efficacy, and safety of IVIG therapy. BT595 provides patients with clinically relevant levels of IgG subclasses to support infection prevention. In this trial, both the Q3W and Q4W dosing regimens achieved adequate serum concentrations of IgG subclasses 1–4 and total IgG.

## Conclusion

In summary, this trial provided a comprehensive and contemporary characterization of IgG subclass pharmacokinetics for BT595, addressing a notable gap in the IVIG literature. BT595 demonstrated PK properties that are fully consistent with established IVIG products, including rapid systemic availability, robust subclass exposure, and maintenance of guideline-relevant total IgG trough concentrations under both Q3W and Q4W dosing regimens. IgG subclass trough concentrations, distribution patterns, maximum exposure, and systemic exposure aligned closely with physiological composition and published IVIG data, without evidence of selective loss or reduced exposure of any subclass.

Notably, under monthly dosing, BT595 exhibited numerically prolonged half-life for certain subclasses, particularly IgG2 and IgG4, suggesting sustained systemic persistence that may support effective maintenance of protective IgG levels over extended dosing intervals. While these observations should be interpreted cautiously due to inter-study variability and methodological differences, they support the suitability of BT595 as an IVIG preparation providing IgG subclass exposure consistent with both clinical practice and endogenous IgG physiology.

Overall, BT595 provides sustained IgG subclass exposure at steady state, supporting its suitability as IVIG replacement therapy in PID under both Q3W and Q4W regimens.
